# Condie on Children

**Published:** 1853-10

**Authors:** 


### CONDIE ON CHILDREN.

It is a just compliment to the talents and industry evinced by Dr. Condie in his treatise on Children, that a second edition has so soon been demanded. We are indebted to Messrs. Blanchard & Lea for a copy, which we have just received at the hands of Mr. Henry C. Morton, bookseller, of this city. It were needless to say that the publishers have discharged their duty, as usual, or to repeat that the work is one of the most practical and judicious that has issued from their prolific press. We recommend every young physician about purchasing a library to embrace in it a copy of this work, and every practitioner, of whatever age, to read it.

